# Double rupture of interventricular septum and free wall of the left ventricle, as a mechanical complication of acute myocardial infarction: a case report

**DOI:** 10.1186/1752-1947-2-85

**Published:** 2008-03-17

**Authors:** Elias I Rentoukas, George A Lazaros, Andreas P Kaoukis, Evangellos P Matsakas

**Affiliations:** 1 Cardiology Department, Athens General Hospital, Athens, Greece

## Abstract

**Introduction:**

Cardiac ruptures following acute myocardial infarction include rupture of the left ventricle free-wall, ventricular septal defects, and papillary muscle rupture. Double myocardial rupture is a rare complication of acute myocardial infarction (0.3 %) and the report of such cases is exclusively limited to a small series of autopsy studies.

**Case presentation:**

In this report we present the unusual case of a 70-year-old woman with acute anteroseptal myocardial infarction, which was complicated by a combined rupture of the interventricular septum near the apex, and the free wall of the left ventricle with concomitant formation of a pseudoaneurysm. The double myocardial rupture was accidentally discovered 10 days later with echocardiography, when the patient, complaining only of mild exertional dyspnea, was hospitalized for a scheduled coronary angiography. The patient underwent successful surgical correction of the double myocardial rupture along with by-pass grafting.

**Conclusion:**

This report highlights the importance of comprehensive noninvasive predischarge diagnostic evaluation of all postinfarct patients, since serious and potentially life-threatening complications might have not been suspected on clinical grounds.

## Introduction

Cardiac ruptures are serious and life-threatening mechanical complications of acute myocardial infarction (AMI). Types of rupture include left ventricle (LV) free-wall rupture (FWR), ventricular septal defect (VSD), and papillary muscle rupture (PMR). Double myocardial rupture (DMR) is defined as the coexistence of two of the above-mentioned forms of rupture. It complicates approximately 0.3% of AMI with the most frequent combination being FWR and VSD [1]. Small autopsy series report that DMR is seen in 13% of patients with FWR and in approximately 16% of patients with VSD [1]. The contribution of 2-D echocardiography and color Doppler in the early diagnosis of these lesions is well established [2]. Since DMR carries a high mortality, surgical correction, even in advanced age, constitutes the treatment of choice [3].

We present the case of a female patient whose recent AMI was complicated by a combination of VSD and FWR of the LV with formation of a pseudoaneurysm, which were successfully surgically corrected. This case is interesting due to the scarcity of such reports and the authors wish to emphasize both the contribution of echocardiography in identifying the above complications and the favorable outcome of our surgically treated patient, despite the seriousness of this complication and its relatively late diagnosis.

## Case presentation

A 70-year-old-female, with a history of diabetes, arterial hypertension and mild chronic renal failure, fifteen days before her admission to our Department, had been admitted to another hospital, because of substernal squeezing pain of ten hours duration and an electrocardiogram compatible with acute anteroseptal myocardial infarction (ST-segment elevation in leads V_1 _to V_4_). Moreover, an echocardiographic study on admission was reported to show regional wall motion abnormalities in the territory of distribution of the left anterior descending coronary artery (LAD). In the absence of contraindications, she was administered fibrinolysis with Tenekteplase, which was considered successful using the current clinical and electrocardiographic criteria. On hospital day 5, the patient had an episode of hypotension, which was treated with infusion of normal saline but no further investigation due to its short duration and her relatively prompt recovery. On the 9^th ^post-infarct day, the patient was discharged with the recommendation for follow-up coronary angiography.

**Figure 1:**
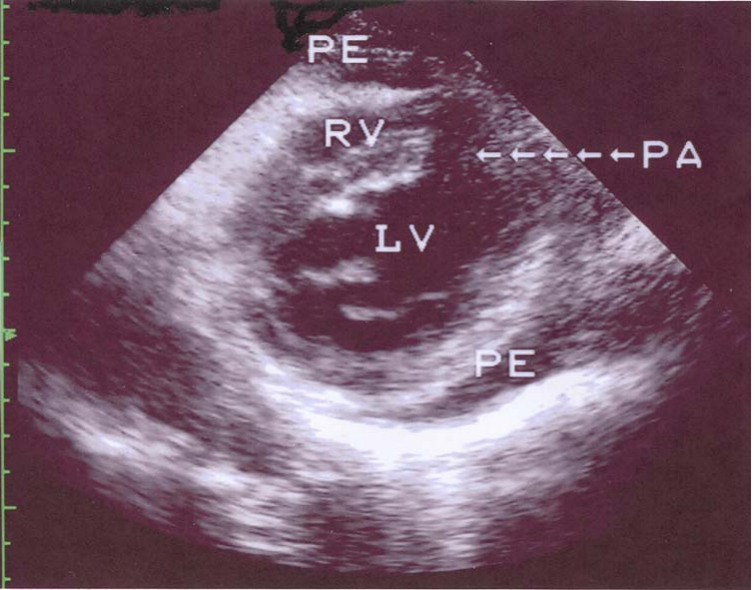
**Modified left parasternal short axis view that shows a discontinuity of the apical part of the interventricular septum and the LV apex, a communication between the left and the right ventricle, and a small cavity contained by epicardium (pseudoaneurysm) through a narrow neck.** (LV: left ventricle, RV: right ventricle, PA: pseudoaneurysm, PE: pericardial effusion).

**Figure 2:**
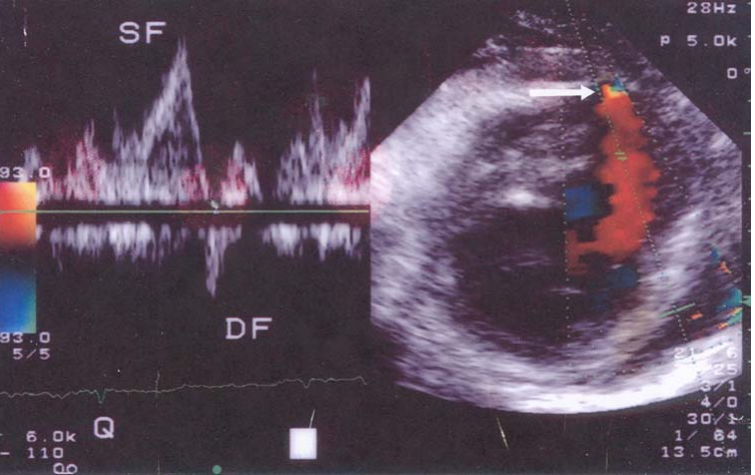
**Pulsed wave Doppler showing a systolic flow (SF) from the LV cavity to the pseudoaneurysm and a diastolic regurgitant flow (DF) in the opposite direction.** In the right part of the picture, colour Doppler depicts a flow between right and left ventricle (white arrow).

**Figure 3:**
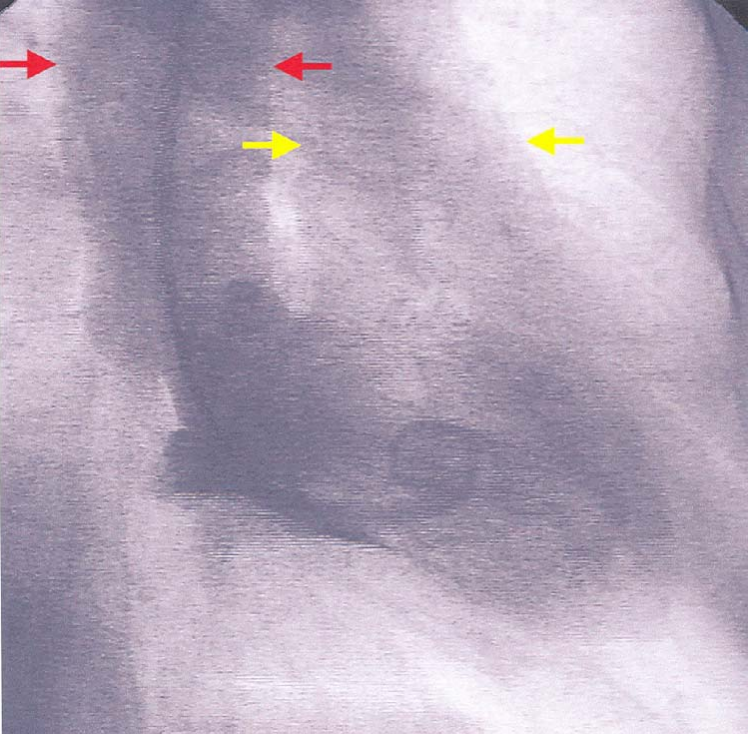
Right anterior oblique left venticulography during systole showing simultaneous opacification of the aorta (red arrows) and the pulmonary artery (yellow arrows).

Six days later, the patient was admitted to our Department for the scheduled coronary arteriography. She reported mild exertional dyspnea and fatigue until about 3 days ago. On examination the patient was an obese woman who appeared well. Her blood pressure was 115/80 and her pulse 80. The only remarkable finding on chest examination was a grade 1-2/6 parasternal holosystolic murmur without gallop or rub. The electrocardiogram was compatible with her recent anteroseptal infarction. An echocardiographic study was performed and disclosed a DMR, consisting of VSD [due to the rupture of the interventricular septum (IVS), with a maximal pressure gradient of approximately 90 mmHg], and rupture of the apical part of the LV free wall with pseudoaneurysm formation (Fig. 1 and 2). The global LV contractility was affected with the ejection fraction being approximately 40%, whereas the anterior wall appeared hypokinetic and the apex akinetic. A coronary arteriography performed on the same day showed a total occlusion of the LAD branch in its proximal part along with an 80% stenosis of the first obtuse marginal branch of the left circumflex coronary artery. The right coronary angiogram disclosed a 50% stenosis in the midportion of the right coronary artery. In addition left ventriculography confirmed the abnormal communication between left and right ventricle (Fig. 3).

On the next day, the patient was transferred to a Cardiac Surgery Center, and underwent surgical closure of the DMR along with double bypass-grafting (left internal mammary artery grafting applied to the LAD and saphenous vein bypass grafting to the obtuse marginal branch).

## Discussion

VSD complicates 1–2% of all AMIs and approximately 0.2 % of fibrinolysed AMIs. In the later case it is seen earlier in the post infarct period (within the first 24 hours or so) in contrast with the non-fibrinolysed AMIs, where it is commonly seen after two to five days [4]. VSD is more common in females, and those with advanced age, anterior AMI and single-vessel disease with poorly developed collaterals to the IVS [4]. In cases of anterior transmural AMI, the rupture is usually located in the anteroapical part of IVS, whilst in inferior infarctions the defect occurs in its basal part [5]. The complication of VSD carries a high mortality and early surgical closure is the treatment of choice, even if the patient's condition is stable. Surgical mortality is high among patients with inferior AMIs (58%), as compared to that of anterior AMIs (25%) [6]. Sporadic reports have also shown that, in selected severely sick and haemodynamically unstable patients with large defects (and consequently shunts), either percutaneous transcatheter closure of the defect or insertion of a left ventricular assist device may improve clinical condition and allow a subsequent surgical repair under better hemodynamics and more favourable local conditions [7,8].

FWR occurs 10 times more frequently than VSD or PMR. Its incidence is higher among patients subjected to late fibrinolysis (i.e. several hours after the onset of symptoms), in comparison to those with early administration of fibrinolysis (within 6–8 hours of the onset of symptoms) [9]. Higher rates of FWR have been also observed in patients taking anti-inflammatory agents (steroids or non-steroidal) [9]. Most patients present with electromechanical dissociation and sudden death or, less frequently, with cardiac tamponade. Some patients may have a subacute course as a result of a contained rupture with pseudoaneurysm formation. In this case there are symptoms of pulmonary congestion, recurrent tachyarrhythmias or systemic embolism. Occasionally, patients may be completely asymptomatic (10–13%) [10]. Spontaneous rupture of a pseudoaneurysm occurs in approximately one third of patients with FWR (as opposed to true LV aneurysms where rupture is quite uncommon), and as a result, surgical resection is recommended regardless of the symptoms or the size of the pseudoaneurysm [11].

DMR is defined as the combination of any two of the three forms of cardiac rupture, with VSD and FWR being the most common (in 17% of patients with VSD there is concomitant FWR) [12]. Tanaka et al. studied a series of ten patients with DMR and concluded that advanced age (mean age 69 years), absence of history of coronary artery disease (90%), anterior AMI (60%), arterial hypertension (60%), and male sex (male/female ratio:8/2) were risk factors for the development of this complication [1]. There are two forms of DMR: a) true, with rupture of both IVS and LV free wall, and b) junctional, located at the junction between IVS and free wall [13]. The analysis of similar cases has shown that the coexistence of FWR is frequently established only at the time of operation for the correction of VSD [3]. Tanaka et al. reported that the majority of patients had an apical AMI and VSD near the junction between IVS and LV apical free wall and concluded that this combination might be a precursor of DMR [1].

Two-dimensional echocardiography, in combination with Doppler study, being an accessible and non-invasive method, contributes significantly both to the diagnosis of every form of cardiac rupture, and the determination of the size of the defect and the magnitude of the left-to-right shunt (as far as VSD is concerned) [2]. Magnetic resonance imaging (MRI) is also a useful tool for the confirmation of diagnosis, particularly when there is a pseudoaneurysm.

Before the 1980s, there was a vogue for managing patients with cardiac rupture non-surgically in the first instant. After a period of perhaps six weeks, often with intraaortic balloon counterpulsation support, the patient underwent surgery. The main advantage of this strategy for the surgeon was that the remaining septum was no longer mushy necrotic muscle, but it had begun to fibrose and thus, was more receptive to sutures. However, the literature in the late 1970s and early 1980s established that there was no place for procrastination, as the great majority of patients died while waiting for the surgical procedure and the mood shifted to early surgical correction of every form of cardiac rupture (including DMR), even in hemodynamically stable patients [14]. Conservative measures such as diuretics, inotropes, nitroprusside and intraaortic balloon counterpulsation are used for the initial stabilization of these patients, as a bridge to surgery. Inferior AMI, right ventricular dysfunction, cardiogenic shock, advanced age, and delay of surgery, are all considered as intraoperative risk factors [6]. In the GUSTO-I study, the 30 day-mortality of surgically managed patients with VSD was 47% as opposed to 94% of conservatively managed patients [4]. Surgical correction of DMR is also accompanied by a high mortality (Tanaka et al. report a 4 month-survival of 37.5%), which nonetheless is less than the mortality of conservative treatment [1]. In the international medical literature, there are mostly case reports of successful surgical correction of DMR, while studies comparing surgical with conservative management have not been performed [3].

The most possible scenario concerning our patient is that the anteroseptal AMI was complicated by VSD near the LV apex. The episode of hypotension at the fifth post-infarct day was probably the manifestation of the second cardiac rupture (FWR), which was easily managed as it resulted in pseudoaneurysm formation without extensive hemopericardium and tamponade. It was quite impressive that the pseudoaneurysm did not rupture during the following ten day period and that the patient, despite the seriousness of this complication, had only mild symptoms. In addition, the detection of DMR was virtually accidental.

## Conclusion

This report emphasizes both the significance and the necessity of the detailed non-invasive evaluation (such as echocardiographic study), in all post-infarct patients, as it may sometimes reveal serious complications that have not been suspected on clinical grounds. Routine pre-discharge echocardiographic evaluation seems also to be a cost-effective approach, as it provides unique information that can significantly impact on patient management decisions.

## Abbreviations

AMI: acute myocardial infarction; LV: left ventricle; FWR: free-wall rupture; VSD: ventricular septal defect; PMR: papillary muscle rupture; DMR: double myocardial rupture; LAD: left anterior descending coronary artery; RV: right ventricle; PA: pseudoaneurysm; PE: pericardial effusion; SF: systolic flow; DF diastolic flow.

## Competing interests

The author(s) declare that they have no competing interests.

## Authors' contributions

EIR was involved in the conception and final reviewing of this report. GAL was involved in the manuscript preparation, editing, and submission. APK was involved in the literature review and manuscript preparation. EPM was involved in the patient's evaluation. All authors read and approved the final manuscript.

## Consent

Written informed consent was obtained from the patient for publication of this case report and accompanying images. A copy of the written consent is available for review by the Editor-in-Chief of this journal.
